# Assessment of the University Students' Knowledge, Attitudes, and Use of Protein Supplements: A Cross-Sectional Study From the United Arab Emirates

**DOI:** 10.1155/jnme/5582105

**Published:** 2025-11-21

**Authors:** Taima Qudah, Sehar Iqbal, Hamza Al Absi, Sayed Sanal, Aya Elkoumi, Leen Fino, Suhad Abumweis

**Affiliations:** ^1^Department of Clinical Pharmacy and AAU Health and Biomedical Research Center, College of Pharmacy, Al Ain University, Abu Dhabi, UAE; ^2^Department of Clinical Pharmacy and Therapeutics, Faculty of Pharmacy, Applied Science Private University, Amman, Jordan; ^3^Department of Clinical Nutrition and Dietetics, Faculty of Applied Medical Sciences, The Hashemite University, Zarqa, Jordan

**Keywords:** cross-section, knowledge, protein supplements, students, UAE

## Abstract

**Background:**

Dietary supplements are widely used by athletes and sportspersons; however, little is known about protein supplement intake among university students undertaking strength training in gyms in the United Arab Emirates (UAE).

**Purpose:**

The purpose of this study was to examine the consumption, knowledge, perceptions, and use of protein supplementation, alone or in association with other supplements, among university students attending fitness centers in the UAE.

**Method:**

A cross-sectional study was conducted among university students in the UAE using a previously validated, self-administered online survey.

**Result:**

A total of 402 adults participated in the study. Approximately 260 (64%) of the participants visited fitness centers, with a higher percentage of males (154/207, 74.4%) than females (106/195, 54.4%). Those who frequented gyms consumed more protein supplements (91/260, 35.0%) compared with nonattendees (8/142, 5.6%). Significant gender differences were observed regarding the benefits and risks of protein supplementation. Among all the participants, 123 (31%) thought that the greatest benefit was building muscle mass, while 141 (35%) believed there was no risk involved. Among protein supplement users, 65 of 109 (59.6%) experienced some type of side effect. Additionally, 57 (14.2%) reported that friends or family encouraged them to take protein supplements.

**Conclusion:**

There is an urgent need to educate university students about the responsible use of protein supplements and the potential risks associated with their misuse. This can be achieved by providing reliable information through university health centers, academic tutors, nutritionists, and awareness campaigns conducted by student wellness services.

## 1. Introduction

Dietary supplements (DS) are bioactive substances designed to complement the diet. These supplements come in various forms including capsules, pills, liquids, powders, and bars, containing ingredients, such as vitamins, minerals, amino acids, enzymes, and herbs [1]. There is substantial evidence worldwide showing the widespread use of DS across various countries. Among the different types of nutritional supplements, protein supplements have become very popular, especially among populations that value fitness [2–5].

In the United Arab Emirates (UAE), many studies have shown that the consumption of DS is becoming increasingly prevalent, particularly among fitness center attendees and university students. For instance, DS consumption was found to be quite prevalent in the UAE, according to a general population survey [6]. In a survey carried out in Sharjah, UAE, among gym attendees, a significant portion of young adult males who frequently visit fitness centers take nutritional supplements; almost 54.3% of male participants use protein powder as their main supplement [7]. Similarly, 39% of participants in research conducted among Ajman University students reported using DS [8]. Additionally, 35.6% of college students in the UAE utilized DS, according to a survey conducted among them. Male students (29.4%) tended to take protein supplements, while female students were more likely to use multivitamins and mineral supplements [9].

International reports have reported similar results. Research conducted in Palermo, Italy, for instance, found that 30.1% of frequent gym-goers took protein supplements, primarily whey protein, frequently in combination with creatine and amino acids. Few users consulted doctors, and none consulted nutritionists; instead, the majority relied on guidance from gym instructors. This illustrates a global trend where informal sources, rather than evidence-based recommendations, are influencing supplement use [10].

Gender differences become particularly evident, when considering the use of protein supplements. Literature also suggested that males are more likely than females to take supplements regularly. For example, it was reported that 42% of U.S. military personnel consume proteins or amino acids, and female gender was one of the factors independently linked to the use of DS (OR female/male: 1.91; 95% CI: 1.73, 2.11) [11]. According to research, males are more likely than females to take protein supplements in an effort to gain muscle mass, boost strength, and improve athletic performance. In contrast, female students tend to use these supplements for purposes, such as weight management, health improvement, and postexercise recovery [3, 10, 12, 13]. This disparity between genders in supplement use reasons is also evident in the UAE, where male university students are more focused on muscle protein synthesis (MPS) and frequently choose whey protein because of its quick absorption and beneficial amino acid composition [7].

Even though protein supplements are widely used, particularly among those who participate in gym-based activities [3, 14, 15], there is a critical gap in the understanding students' attitudes, knowledge, and usage habits of these supplements. Many students are unaware of the risks associated with improper use of supplements and may rely on peer advice or social media influencers for guidance instead of formal nutritional education [15–18]. Moreover, there is little research specifically addressing the factors influencing protein supplement use among university students in the UAE.

Given the growing popularity of fitness culture and the growing dependence on DS among UAE university students, there is a critical gap in the literature about the specific knowledge, attitudes, and use of protein supplements in this group of people. While there are studies on supplement use in general, few have focused particularly on university students in the UAE. Thus, this study aims to fill this gap by exploring how male and female students in the UAE differ in their reasons for using protein supplements, the sources of information that influence their decisions, and their understanding of the potential risks. These insights the development of educational interventions to help students make well-informed decisions about supplement use.

## 2. Method

### 2.1. Ethical Considerations

The study received the required ethical approval from the Research Ethics Commission (REC) at Al Ain University (reference number: COP/AREC/AD/09).

### 2.2. Study Design and Sampling

A cross-sectional online survey was created and circulated to UAE university students from November 2023 to April 2024 to evaluate their knowledge, attitude, and use of protein supplements. The questionnaire was uploaded to Google Forms, an online survey platform. Snowball sampling recruitment techniques were used to recruit participants online. The survey link was first made available to university students via WhatsApp and other social media channels. The invitation included a brief overview of the study. Participants were informed that the survey was anonymous, with no collection of geolocation data or personally identifiable information, and that their responses would remain confidential. Participation was entirely voluntary, and participants could withdraw from the questionnaire at any time. The survey did not ask for names or any other identifying details, and participants were free to exit the survey by closing the browser at any point. Based on the RaoSoft calculator—with a 5% margin of error, 95% confidence level, a population size of 10 million (representing the UAE) [19], and a 50% response distribution—a minimum sample size of 385 participants was recommended, consistent with previous studies targeting a similar population [20]. This study ultimately included 402 university student respondents.

### 2.3. Study Tool

A comprehensive literature search using web-based databases and relevant research served as the foundation for the development of a validated questionnaire [21, 22]. The instrument's validity and its comparability to those polls were key considerations when choosing which questions to include. In addition, the study team assessed each draft of the questionnaire separately to make sure it was valid and applicable to university students in the UAE. Additionally, 15 students participated in an online pilot test of the study, and the pilot data were not included in the final analysis. The experts' feedback and the outcomes of the pilot test were used to modify the study instrument. A final version was made available and was applied to the data gathering. The full survey instrument is provided in Supporting File 1.

The questionnaire was divided into two sections. Section one: nineteen questions about sociodemographic characteristics, such as age and gender, gym attendance, protein consumption, supplement use and timing and reasons for use, and symptoms related to protein intake. Participants' knowledge regarding the advantages and disadvantages of protein supplements and the source of information influencing their decision were also assessed. Section two: Eight statements regarding protein supplements were presented to the respondents, and they were asked to select their responses on a 5-point Likert scale (strongly agree, agree, neutral, disagree, and strongly disagree).

The primary outcome variable was the self-reported use of protein supplements (yes/no). Key explanatory variables included gender (male/female), age category (18–25 years, 26–30 years, and above 30+), and gym attendance frequency (none, 1–2 per week, 3–5 per week, and more than 5.).

### 2.4. Data Analysis

Version 26 of the Statistical Package for the Social Sciences (SPSS) was used to analyze the data. For every variable, descriptive data were acquired, and the differences by sex, protein supplement use, and gym attendance were assessed using cross-tabulation and chi-square testing. For ordered categorical variables, such as the 5-point Likert scale responses, the Mann–Whitney U-test was applied to assess differences between groups. A value of ≤ 0.05 was designated as the level of significance.

All survey responses were mandatory; hence, the final dataset contained no missing data. Questionnaires with partial or incomplete responses were not included in the study. The snowball sampling technique was employed in this investigation without any weighting or sampling probability correction. Due to the study's primary descriptive and exploratory nature, no sensitivity analyses were conducted.

## 3. Results

A total of 402 participants—207 (51.4%) males and 195 (48.5%) females—agreed to take part in the study. Descriptive characteristics by sex are presented in Table 1. Almost 88% of the sample was aged between 18 and 25, and 260 (64.7%) of participants were fitness center attendees. Compared to females (106/260, 54.4%), a higher proportion of males (154/260, 74.4%, *p* < 0.001) signed up for the gym. Males went to the gym more frequently than females did as well 156 (75.4%) vs. 122 (62.6%). The majority of fitness center attendees (130, 32.4% of the overall sample) went to the gym three to five times per week.

When asked whether they calculate their protein requirements, 63 of the 207 male respondents (30.4%) said yes, while 37 of the 195 female respondents (19.0%) said yes. This difference was statistically significant (*p*=0.021). In terms of protein supplement consumption, males (58, 28%) consumed significantly more than females (41, 21%) (*p*=0.027).

The amount and timing of protein supplement consumption by gender are shown in Table 2. Protein powder was the most popular supplement among male users (58/147, 39.5%). In contrast, female participants preferred snacks, such as brownies, pancakes, cookies, and chips (49/117, 41.9%), over other protein supplements. One scoop of protein supplements per day was the most commonly consumed among protein powder users (39/90, 43.3%) usually taken immediately after exercise (85/220, 38.6%). The most frequent amount of protein powder consumed was about 24 g/day.

Furthermore, 65 (59.6%) of individuals consuming protein supplements experienced side effects as a result of their supplementation, primarily gastrointestinal (constipation, diarrhea, indigestion, nausea, and appetite loss [36, 33%]). Following that, 20 (18.3%) of the participants experienced headache, insomnia, and stress, while 9 (8.3%) reported symptoms, such as skin rashes and itching.

The cross-tabulation of protein supplement consumption and gym attendance is shown in Table 3. When comparing by gender, both males and females who attended the gym reported higher use than nonattendees (*p* < 0.001). Among males, (53/154, 34.4%) gym attendees used supplements compared with (5/53, 9.4%) nonattendees. Among females, 38 of 106 (35.8%) attendees used supplements compared with three of 89 (3.4%) nonattendees. Overall, 91 participants (35.0%) who regularly attended the gym reported taking protein supplements.

Table 4 displays the rationale, benefits, and disadvantages of protein supplements based on gender. There were no significant gender differences (*p*=0.378) in the top two reasons for supplement use: 42 of 99 (42.3%) to build a muscular body and 24 of 99 (24.3%) to gain muscle strength. The results also revealed that 57 (14.2%) of the participants reported that friends or family had encouraged them to use protein supplements, while 35 (8.7%) of the participants overall reported that their coaches had encouraged them to do so. Furthermore, about a third of the participants were aware of the risks involved in taking protein supplements, but they were unable to identify them. More males (93/207, 45.0%) than females (48/195, 24.6%) believed that taking protein supplements carries no risk at all (141, 35.1%). For 123 (31%) of the sample, the main perceived benefit was building muscle mass.

Table 5 presents participants' knowledge and attitudes toward protein supplements. The majority of participants seemed to be in agreement or strongly in agreement with the statements that protein supplements are a good source of energy for exercisers, necessary for weightlifters to build muscle, a good way to gain more muscle, and eating large amounts of protein will negatively affect an individual's health. However, almost half of the participants disagreed or strongly disagreed that taking protein supplements reduces undesired body fat from accumulating. Participants were mainly in disagreement with the statement that protein supplements are superior to foods rich in protein for building muscle.

## 4. Discussion

This cross-sectional survey assessed the use of protein supplements among university students and looked at the attitudes and knowledge of both male and female students regarding these supplements. Our finding shows a higher tendency of protein supplement intake in males as compared to females. Also, the consumption of protein powder was much higher in males compared with females due to the belief that it is necessary for muscle building and its perceived ergogenic effects.

Previous studies support these findings. For example, males (47.7%) consumed more DS than females (28.1%) in a previous local study on people who use gyms in a university community in Sharjah, UAE [7]. Similarly, male Saudi gym attendees were found to consume protein supplements at a higher rate (68.7%) than female attendees (35.6%) [21]. Internationally, males are generally more likely than females to consume supplements [2, 3, 23]. In Spain, among 415 fitness center users, 42.7% of males and only 3.2% of females reported protein powder use [24]. In Canada, male athletes were more likely than female athletes to consume energy drinks, beta-alanine, branched-chain amino acids, glutamine, and protein powder [15]. However, among groups of European endurance runners, gender was not found to be a significant modulator of supplement intake [25].

Our participants, who were mainly between 18 and 25 years old, were mainly encouraged by a relative or a friend to take protein supplements. Peer influence in young adults is known to affect many of their behaviors [17, 18, 26]. Other studies indicate that protein supplement users often rely on Internet sources and coaches for information [21, 27].

Protein intake after endurance exercise is known to increase MPS [28]. Our participants mainly consumed protein supplements to build a muscular body consistent with prior studies [21, 27]. However, clinical trials show that 30 g of high-quality dietary protein is sufficient to support MPS after endurance exercise [28], and ingestion of ≥ 1.6 g of protein/kg/day produces only small lean muscle mass gain in adults under 65 [29]. Thus, the use of protein supplements to enhance muscle building may be an unnecessary cost for young people.

The most frequent amount of protein powder consumed by our participants was about 24 g/day. Similar to our study, Alhakbany et al. reported that 42.6% of the participants in his study used 24 g/day of protein supplements [21]. Evidence suggests that 20–30 g of protein during or after exercise improves nitrogen balance and increases MPS [30]. Higher intakes (> 40 g) will not further increase MPS [31]. Consuming protein during the recovery phase after resistance exercise contributes to an increase in strength and skeletal MPS [32]. In this regard, essential amino acids (mostly found in protein supplements) help to regulate protein metabolism in muscles, promote anabolic pathways in athletes, mitigate cachexia in muscle-wasting disorders, lessen exercise-induced fatigue, accelerate the healing of wounds, and increase the production of insulin [33]. The increase in protein supplements among young adults necessitates proper education about the needed daily amount to promote muscle synthesis after exercising especially since a high intake of DS can cause some adverse events [31, 34].

Participants of this study reported a high incidence of GIT symptoms (constipation, diarrhea, indigestion, nausea, and lack of appetite) followed by headache, insomnia, and stress while using protein supplements. Similarly, few other studies reported the side effects of protein supplements including abnormal heartbeats, stomach pain, dizziness, tremors, and dizziness [35, 36]. A precursor pool of short-chain fatty acids is stimulated in the large intestine by the microbial fermentation of various amino acids, yields branched-chain fatty acids, and appears to alter the GI microbiota's composition, metabolism, and energy homeostasis [31].

The presence of pharmacological substances, microorganism adulteration, and the presence of toxic metals are other factors leading to adverse health effects of supplement intake. Also, prohormones with or without being listed on the ingredient label might disturb the natural hormonal balance, abnormal growth, or sex development in adolescents and children along with other undesired side effects in adults [37]. Moreover, a high protein (amino acids) intake increases ammonia production in individuals with already compromised hepatic or renal issues and may disturb amino acid homeostasis and insulin metabolism [38].

Regarding knowledge and attitude, most of our study participants failed to identify the precise risks connected with taking protein supplements and believed that protein supplements are better than protein-rich food for building muscles and their ergogenic effects on the body. As MPS mainly depends on the intake after exercise and type of the training, this misconception suggests that students may not fully understand how protein and exercise work together to support muscle growth [31]. Social media also contributes to misinformation and influences supplement use [39]. Similarly, the consciousness of physical appearance and exposure to fitness-related content posted on social media is positively associated with the consumption of DS and steroid intake in the young male population [40]. Proper education can improve the university students' knowledge about protein supplements [41].

In line with these concerns, recent studies have reported that university students in the UAE are using performance-enhancing drugs (PEDs) and cognitive enhancers, even though they are prohibited. According to a quantitative study by Sharif et al., a sizable portion of students knew about and had access to cognitive enhancers designed to improve academic performance [20]. More information about student attitudes, motives, and the sources of these substances was revealed by their later qualitative study [42]. These results support our findings that peer pressure, social goals, and misinformation have an impact on protein supplement use, and they also highlight the larger trend of performance-driven behaviors among students, whether they be cognitive or physical. Promoting safe and knowledgeable decision-making among university populations requires addressing these interrelated behaviors through integrated health education and awareness initiatives.

Literature illustrated that a healthy lifestyle, balanced food intake, and daily physical activity ameliorate the global disease burden [43]. During workouts, the body and metabolic requirements increase due to high-intensity physical activities. Therefore, a high intake of protein for people engaged in regular physical activity (i.e., 1.4–2.0 g/kg/day) is suggested to improve training, immune function, growth, and maintenance of lean body mass. Media-driven self-prescribed protein supplementation is common, often without any professional guidance [36, 40]. In addition, the easy access to high-protein products, such as protein powder, energy drinks, DS, protein bars, sports drinks and gels, and noncarbonated, noncaffeinated sports beverages, is making the situation more complicated [44]. Professional guidelines recommended protein intake of 1.2–1.7 g/kg/day for general athletic performance, 1.5–2.0 g protein/kg/day for strength athletes, and 1.83 g protein/kg/day for endurance athletes [45, 46]. A varied diet can usually meet protein requirements without supplementation [47].

## 5. Study Limitations

This study evaluated the consumption patterns, knowledge, and perception of protein supplements among university students; however, our study has a few limitations. The use of self-reported questionnaires to collect data may introduce reporting bias. Additionally, the snowball sampling technique could increase the risk of selection bias, as participants may recruit others with similar characteristics. While the large sample size improves the precision of our estimates and reduces the effect of random variation, it does not eliminate the risk of selection bias. Therefore, caution is needed when generalizing the findings to the broader population. In addition, it has been limited by social desirability bias, which we aimed to control through anonymity. Anonymity is considered to lower social desirability bias and social anxiety. Moreover, the questionnaire did not include items assessing the sources of information regarding the potential negative effects of protein supplement use (e.g., healthcare professionals, university staff, family members, or media). Including such questions could have provided deeper insight into the factors influencing students' knowledge and attitudes toward supplement use. Another limitation of this study is the absence of multivariable analyses (e.g., logistic regression). Given the use of snowball sampling, the sample is prone to selection bias, and conducting such analyses could have produced misleading associations between explanatory variables. Future studies should employ probability-based sampling strategies and incorporate multivariable analyses to allow for more robust and generalizable findings. Lastly, this study did not inquire about students' use of androgens or other PEDs, which are illegal in the UAE and could therefore be underreported.

## 6. Conclusion and Recommendations

Our study found a high consumption of protein supplements among males as compared to females. Regarding knowledge and perception, most of the participants did not know about the health risks associated with excessive protein supplements. Also, participants strongly believed in taking protein supplements to fulfill energy requirements while doing physical activity.

Current guidelines are mostly unclear and inconsistent regarding the recommended intake of protein supplements during workouts. The message communicated to the young population about the role of dietary protein and supplementations in managing weight and body composition is often complex and contradictory for safe dietary intake. Future policy recommendations should be evidence-based and adapted to the requirements of young adults who engage in physical activity. Academic programs, student wellness services, and university health centers should all be used to execute educational techniques. These could include peer-led lectures, organized workshops run by certified dietitians, and awareness campaigns conducted online and on campus. Giving students accurate information about their daily protein requirements, the potential negative effects of excessive ingestion, and the limited advantages of supplementing when not medically recommended should be the goal. The relationship between actual protein requirements, present consumption habits, and related health hazards must also be investigated further. These observations will be useful in directing future regulations and enhancing the appropriate usage of protein supplements by various demographic groups.

## Figures and Tables

**Table 1:** Descriptive characteristics of the participants relative to gender (*N* (%)).

Variable	All	Males	Females	*p* value
	*n* = 402	*n* = 207	*n* = 195	
Age category				
18–25 years	353 (87.9%)	184 (88.9%)	169 (86.7%)	0.943
26–30 years	24 (5.9%)	11 (5.3%)	13 (6.7%)	
Above 30+	25 (6.2%)	12 (5.8%)	13 (6.7%)	
Do you regularly attend the gym?				
Yes	260 (64.7%)	154 (74.4%)	106 (54.4%)	< 0.001
No	142 (35.3%)	53 (25.6%)	89 (45.6%)	
Gym visits per week?				
I don't go to the gym.	124 (30.8%)	51 (24.6%)	73 (37.4%)	0.399
1–2 per week	56 (13.9%)	28 (13.5%)	28 (14.4%)	
3–5 per week	130 (32.4%)	71 (34.4%)	59 (30.3%)	
More than 5	92 (22.9%)	57 (27.5%)	35 (18.0%)	
Do you calculate your daily protein requirements?				
Yes	100 (24.8%)	63 (30.4%)	37 (19.0%)	0.021
No	302 (75.2%)	144 (69.6%)	158 (81.0%)	
Do you consume any protein supplements?				
Yes	99 (24.6%)	58 (28.0%)	41 (21.0%)	0.027
No	303 (75.4%)	149 (72.0%)	154 (79.0%)	

*Note: p* values of the chi-square test.

**Table 2:** The amount, timing, and symptoms of protein supplement consumption relative to use (*N* (%)).

Variable	All	Males	Females	*p* value^∗^
What kind of protein supplement do you consume?^∗∗^	*n* = 264	*n* = 147	*n* = 117	
Powder	90 (34.1%)	58 (39.5%)	32 (27.4%)	0.241
Protein bars	74 (28.0%)	38 (25.8%)	36 (30.7%)	
Other snacks: (brownies, pancakes, cookies, and chips)	100 (37.9%)	51 (34.7%)	49 (41.9%)	
If you are using protein powder, how many scoops (grams) do you consume daily?	*n* = 90	*n* = 58	*n* = 32	
1/2–1 scoop (12–24 g)	22 (24.4%)	12 (20.7%)	10 (31.2%)	0.404
1 scoop (24 g)	39 (43.3%)	24 (41.4%)	15 (46.9%)	
1-2 scoops (24–48 g)	23 (25.6%)	17 (29.3%)	6 (18.8%)	
More than 2 scoops	6 (6.7%)	5 (8.6%)	1 (3.1%)	
When do you take protein supplements?^∗∗^	*n* = 220	*n* = 129	*n* = 91	
Before workout	56 (25.5%)	31 (24.0%)	25 (27.4%)	< 0.001
Early morning	50 (22.7%)	27 (20.9%)	23 (25.3%)	
Right after workout	85 (38.6%)	53 (41.1%)	32 (35.2%)	
Other time	29 (13.2%)	18 (14.0%)	11 (12.1%)	
Symptoms while taking protein supplements.^∗∗^	*n* = 109	*n* = 74	*n* = 44	
Gastrointestinal tract–related symptoms	36 (33.0%)	32 (43.2%)	13 (29.6%)	0.512
Headache, insomnia, and stress	20 (18.3%)	12 (16.2%)	8 (18.2%)	
Skin rash and itching	9 (8.3%)	7 (9.5%)	2 (4.5%)	
No symptoms	33 (30.3%)	16 (21.6%)	17 (38.6%)	
Others	11 (10.1%)	7 (9.5%)	4 (9.1%)	

^∗^ *p* values of the chi-square test.

^∗∗^participants could pick more than one response.

**Table 3:** Cross-tabulation of protein supplement consumption with gym attendance among participants (*N* (%)).

**Gym attendance**	**Protein supplement consumption**			***p* value** ^**∗**^
	**Yes**	**No**		

			Males	
Yes	53 (34.4%)	101 (65.6%)	*n* = 154	
No	5 (9.4%)	48 (90.6%)	*n* = 53	< 0.001
			Females	
Yes	38 (35.8%)	68 (64.2%)	*n* = 106	
No	3 (3.4%)	86 (96.6%)	*n* = 89	< 0.001
			All	
Yes	91 (35.0%)	169 (65.0%)	*n* = 260	
No	8 (5.6%)	134 (94.4%)	*n* = 142	< 0.001

^∗^ *p* values of the chi-square test.

**Table 4:** Participants' responses to questions about the reason for taking protein and benefits and risks of protein supplement consumption relative to gender (*N* (%)).

Variable	All	Male	Female	*p* value^∗^
For what reason do you take protein supplements?^∗∗^	*n* = 99	*n* = 58	*n* = 41	
Building a muscular body	42 (42.3%)	28 (48.3%)	14 (34.2%)	0.378
Gaining power and strength	24 (24.3%)	14 (24.2%)	10 (24.4%)	
Competitive advantage.	5 (5.1%)	2 (3.4%)	3 (7.3%)	
Improve body shape.	19 (19.2%)	8 (13.8%)	11 (26.8%)	
Others	9 (9.1%)	6 (10.3%)	3 (7.3%)	
Who encouraged you to take protein supplements?	*n* = 402	*n* = 207	*n* = 195	
Friends/relatives	57 (14.2%)	36 (17.4%)	21 (10.8%)	0.001
Coach	35 (8.7%)	18 (8.7%)	17 (8.6%)	
Healthcare provider	15 (3.7%)	7 (3.4%)	8 (4.1%)	
Social media/internet	30 (7.5%)	17 (8.2%)	13 (6.7%)	
Others	22 (5.5%)	13 (6.3%)	9 (4.6%)	
No one	243 (60.4%)	116 (56%)	127 (65.2%)	
Are there risks associated with taking protein supplements?	*n* = 402	*n* = 207	*n* = 195	
No risk	141 (35.1%)	93 (45.0%)	48 (24.6%)	0.002
Yes, but I don't know the risk	126 (31.3%)	53 (25.6%)	73 (37.4%)	
Kidney damage	70 (17.4%)	31 (15%)	39 (20%)	
Dehydration	20 (5.0%)	4 (1.9%)	16 (8.2%)	
Gout (joint inflammation)	7 (1.7%)	4 (1.9%)	3 (1.5%)	
Others	38 (9.5%)	22 (10.6%)	16 (8.2%)	
Are there benefits associated with taking supplements?	*n* = 402	*n* = 207	*n* = 195	
No	19 (4.7%)	8 (3.9%)	11 (5.6%)	0.882
I don't know.	93 (23.0%)	50 (24.2%)	43 (22.1%)	
Yes, but I don't know the exact benefits.	46 (11.4%)	21 (10.1%)	25 (12.8%)	
Building muscular body	123 (30.9%)	65 (31.4%)	58 (29.7%)	
Gaining power and strength	39 (9.7%)	22 (10.6%)	17 (8.7%)	
Improves performance.	67 (16.6%)	33 (15.9%)	34 (17.4%)	
Others	15 (3.7%)	8 (3.9%)	7 (3.6%)	

^∗^ *p* values of the chi-square test.

^∗∗^participants could pick more than one response.

**Table 5:** Knowledge and attitudes of participants about protein supplements relative to gender (*N* (%)).

Variable	All *n* = 402	Males *n* = 207	Females *n* = 195	*p* value^∗^
Knowledge of participants:				
Protein supplements are a good source of energy during exercise.				
I agree	177 (44.1%)	91 (44.0%)	86 (44.1%)	0.022
I strongly agree	50 (12.4%)	18 (8.7%)	32 (16.4%)	
Neutral	123 (30.6%)	66 (31.8%)	57 (29.2%)	
I disagree	41 (10.2%)	26 (12.6%)	15 (7.7%)	
I strongly disagree	11 (2.7%)	6 (2.9%)	5 (2.6%)	
Protein supplements are essential for building muscles through weightlifting				
I agree	188 (46.8%)	95 (45.9%)	93 (47.7%)	0.428
I strongly agree	59 (14.7%)	35 (16.9%)	24 (12.3%)	
Neutral	108 (26.8%)	51 (24.6%)	57 (29.2%)	
I disagree	35 (8.7%)	20 (9.7%)	15 (7.7%)	
I strongly disagree	12 (3.0%)	6 (2.9%)	6 (3.1%)	
Most people my age fulfill their protein needs.				
I agree	176 (43.9%)	82 (39.6%)	94 (48.2%)	0.621
I strongly agree	99 (24.5%)	54 (26.1%)	45 (23.1%)	
Neutral	110 (27.4%)	60 (29.0%)	50 (25.6%)	
I disagree	13 (3.2%)	7 (3.3%)	6 (3.1%)	
I strongly disagree	4 (1.0%)	4 (2.0%)	0 (0%)	
Protein supplements are better than protein-rich food to build muscles				
I agree	64 (16.0%)	38 (18.3%)	26 (13.3%)	0.287
I strongly agree	17 (4.2%)	9 (4.3%)	8 (4.10%)	
Neutral	123 (30.6%)	64 (30.9%)	59 (30.2%)	
I disagree	121 (30.1%)	56 (27.1%)	65 (33.3%)	
I strongly disagree	77 (19.1%)	40 (19.4%)	37 (19.1%)	
Taking protein supplements minimizes the accumulation of unwanted body fat				
I agree	85 (21.2%)	48 (23.2%)	37 (19.0%)	0.658
I strongly agree	30 (7.5%)	17 (8.2%)	13 (6.7%)	
Neutral	191 (47.5%)	84 (40.6%)	107 (54.9%)	
I disagree	76 (18.8%)	43 (20.8%)	33 (16.9%)	
I strongly disagree	20 (5.0%)	15 (7.2%)	5 (2.6%)	
Attitude of participants:				
Gym attendees should take protein supplements.				
I agree	142 (35.3%)	74 (35.7%)	68 (34.9%)	0.193
I strongly agree	45 (11.2%)	27 (13.1%)	18 (9.2%)	
Neutral	154 (38.3%)	78 (37.7%)	76 (39.0%)	
I disagree	47 (11.7%)	22 (10.6%)	25 (12.8%)	
I strongly disagree	14 (3.5%)	6 (2.9%)	8 (4.1%)	
To gain more muscles, I need to take protein supplements				
I agree	155 (38.6%)	82 (39.6%)	73 (37.4%)	0.871
I strongly agree	47 (11.7%)	25 (12.1%)	22 (11.3%)	
Neutral	119 (29.6%)	56 (27.1%)	63 (32.3%)	
I disagree	57 (14.2%)	29 (14.0%)	28 (14.4%)	
I strongly disagree	24 (6.0%)	15 (7.2%)	9 (4.6%)	
Eating large amounts of protein will negatively affect an individual's health				
I agree	162 (40.3%)	81 (39.1%)	81 (41.5%)	0.696
I strongly agree	85 (21.1%)	44 (21.3%)	41 (21.1%)	
Neutral	100 (24.9%)	52 (25.1%)	48 (24.6%)	
I disagree	36 (9.0%)	18 (8.7%)	18 (9.2%)	
I strongly disagree	19 (4.7%)	12 (5.8%)	7 (3.6%)	

^∗^ *p* values of the Mann–Whitney U-test.

## Data Availability

The datasets generated and analyzed during this study are available from the corresponding author upon reasonable request.

## Supporting Information

Supporting File 1: Survey Instrument.

Complete survey used to assess university students' knowledge, attitudes, and use of protein supplements.

## References

[1] https://ods.od.nih.gov/factsheets/WYNTK-Consumer/.

[2] Thomas E., Karsten B., Sahin F. N. (2019). Protein Supplement Consumption is Linked to Time Spent Exercising and high-protein Content Foods: A Multicentric Observational Study. *Heliyon*.

[3] El Khoury D., Antoine-Jonville S. (2012). Intake of Nutritional Supplements Among People Exercising in Gyms in Beirut City. *Journal of Nutrition and Metabolism*.

[4] Bailey R. L., Gahche J. J., Lentino C. V. (2011). Dietary Supplement Use in the United States, 2003–2006. *The Journal of Nutrition*.

[5] Murphy S. P., Wilkens L. R., Monroe K. R. (2011). Dietary Supplement Use Within a Multiethnic Population as Measured by a Unique Inventory Method. *Journal of the American Dietetic Association*.

[6] Kaddoura N. H., AlAhmad M., Hassan N., Alomar M. J. (2022). Dietary Supplements’ Knowledge, Attitude and Consumption Pattern Among United Arab Emirates Population. *British Food Journal*.

[7] Ibrahim Sharif S., Mohammed A., Mohammed I., Suleiman Sharif R. (2018). Evaluation of Knowledge, Attitude and Use of Dietary Supplements Among People Exercising in the Gym in Sharjah-United Arab Emirates. *Physical Medicine and Rehabilitation Research*.

[8] Alhomoud F. K., Basil M., Bondarev A. (2016). Attitudes and Practices (KAP) Relating to Dietary Supplements Among Health Sciences and Non-Health Sciences Students in One of the Universities of United Arab Emirates (UAE). *Journal of Clinical and Diagnostic Research: Journal of Clinical and Diagnostic Research*.

[9] Radwan H., Hasan H. A., Ghanem L. (2019). Prevalence of Dietary Supplement Use and Associated Factors Among College Students in the United Arab Emirates. *Journal of Community Health*.

[10] Bianco A., Mammina C., Paoli A. (2011). Protein Supplementation in Strength and Conditioning Adepts: Knowledge, Dietary Behavior and Practice in Palermo, Italy. *Journal of the International Society of Sports Nutrition*.

[11] Knapik J. J., Trone D. W., Steelman R. A., Farina E. K., Lieberman H. R. (2021). Prevalence of and Factors Associated With Dietary Supplement Use in a Stratified, Random Sample of US Military Personnel: The US Military Dietary Supplement Use Study. *The Journal of Nutrition*.

[12] Knapik J. J., Steelman R. A., Hoedebecke S. S., Austin K. G., Farina E. K., Lieberman H. R. (2016). Prevalence of Dietary Supplement Use by Athletes: Systematic Review and Meta-Analysis. *Sports Medicine*.

[13] Morton R. W., Murphy K. T., McKellar S. R. (2018). A Systematic Review, Meta-Analysis and Meta-Regression of the Effect of Protein Supplementation on Resistance Training-Induced Gains in Muscle Mass and Strength in Healthy Adults. *British Journal of Sports Medicine*.

[14] Slater G., Tan B., Teh K. C. (2003). Dietary Supplementation Practices of Singaporean Athletes. *International Journal of Sport Nutrition and Exercise Metabolism*.

[15] Wiens K., Erdman K. A., Stadnyk M., Parnell J. A. (2014). Dietary Supplement Usage, Motivation, and Education in Young Canadian Athletes. *International Journal of Sport Nutrition and Exercise Metabolism*.

[16] Jacobson B. H., Sobonya C., Ransone J. (2001). Nutrition Practices and Knowledge of College Varsity Athletes: A Follow-Up. *The Journal of Strength & Conditioning Research*.

[17] Smolak L., Murnen S. K., Thompson J. K. (2005). Sociocultural Influences and Muscle Building in Adolescent Boys. *Psychology of Men and Masculinity*.

[18] Henneberger A. K., Mushonga D. R., Preston A. M. (2021). Peer Influence and Adolescent Substance Use: A Systematic Review of Dynamic Social Network Research. *Adolescent Research Review*.

[19] https://www.worldometers.info/world-population/united-arab-emirates-population/.

[20] Sharif S., Fergus S., Guirguis A., Smeeton N., Schifano F. (2022). Assessing Prevalence, Knowledge and Use of Cognitive Enhancers Among University Students in the United Arab Emirates: A Quantitative Study. *PLoS One*.

[21] Alhakbany M. A., Alzamil H. A., Alnazzawi E. (2022). Knowledge, Attitudes, and Use of Protein Supplements Among Saudi Adults: Gender Differences. *Health Care*.

[22] Duellman M. C., Lukaszuk J. M., Prawitz A. D., Brandenburg J. P. (2008). Protein Supplement Users Among High School Athletes Have Misconceptions About Effectiveness. *The Journal of Strength & Conditioning Research*.

[23] Hartmann C., Siegrist M. (2016). Benefit Beliefs About Protein Supplements: A Comparative Study of Users and Non-Users. *Appetite*.

[24] Sánchez Oliver A., Miranda León T., Guerra Hernández E. (2011). Prevalence Ofprotein Supplement Use at Gyms. *Nutricion Hospitalaria*.

[25] Wirnitzer K., Motevalli M., Tanous D. R. (2021). Sex Differences in Supplement Intake in Recreational Endurance Runners—Results From the Nurmi Study (Step 2). *Nutrients*.

[26] McCabe M. P., Ricciardelli L. A., Holt K. (2005). A Longitudinal Study to Explain Strategies to Change Weight and Muscles Among Normal Weight and Overweight Children. *Appetite*.

[27] Wafi A. M., Alhazmi O. A., Jathmi A. J. (2024). Protein Supplement Intake by Non-Athlete Gym Attendees in Jazan Region: Misconceptions and Gender Differences. *The Journal of Sports Medicine and Physical Fitness*.

[28] Churchward-Venne T. A., Pinckaers P. J. M., Smeets J. S. J. (2020). Dose-Response Effects of Dietary Protein on Muscle Protein Synthesis During Recovery From Endurance Exercise in Young Men: A Double-Blind Randomized Trial. *The American Journal of Clinical Nutrition*.

[29] Nunes E. A., Colenso-Semple L., McKellar S. R. (2022). Systematic Review and Meta-analysis of Protein Intake to Support Muscle Mass and Function in Healthy Adults. *Journal of Cachexia, Sarcopenia and Muscle*.

[30] Thomas D. T., Erdman K. A., Burke L. M. (2016). Position of the Academy of Nutrition and Dietetics, Dietitians of Canada, and the American College of Sports Medicine: Nutrition and Athletic Performance. *Journal of the Academy of Nutrition and Dietetics*.

[31] Kårlund A., Gómez-Gallego C., Turpeinen A. M., Palo-Oja O. M., El-Nezami H., Kolehmainen M. (2019). Protein Supplements and Their Relation With Nutrition, Microbiota Composition and Health: Is More Protein Always Better for Sportspeople?. *Nutrients*.

[32] Reidy P. T., Rasmussen B. B. (2016). Role of Ingested Amino Acids and Protein in the Promotion of Resistance Exercise-Induced Muscle Protein Anabolism. *The Journal of Nutrition*.

[33] Holeček M. (2018). Branched-Chain Amino Acids in Health and Disease: Metabolism, Alterations in Blood Plasma, and as Supplements. *Nutrition and Metabolism*.

[34] Jang C., Oh S. F., Wada S. (2016). A Branched-Chain Amino Acid Metabolite Drives Vascular Fatty Acid Transport and Causes Insulin Resistance. *Nature Medicine*.

[35] Austin K. G., Farina E. K., Lieberman H. R. (2016). Self-Reported Side-Effects Associated With Use of Dietary Supplements in an Armed Forces Population. *Drug Testing and Analysis*.

[36] Singhvi B., Gokhale D. (2021). Usage of Nutritional Supplements and Its Side Effects Among Gym Goers in Pune. *Cardiometry*.

[37] Da Costa B. R. B., Roiffé R. R. (2021). Quality Control of Protein Supplements: A Review. *International Journal of Sport Nutrition and Exercise Metabolism*.

[38] Holeček M. (2022). Side Effects of Amino Acid Supplements. *Physiological Research*.

[39] Vasconcelos Q. D. J. S., Bachur T. P. R., Aragão G. F. (2021). Whey Protein Supplementation and Its Potentially Adverse Effects on Health: A Systematic Review. *Applied Physiology Nutrition and Metabolism*.

[40] Hilkens L., Cruyff M., Woertman L., Benjamins J., Evers C. (2021). Social Media, Body Image and Resistance Training: Creating the Perfect ‘Me’ With Dietary Supplements, Anabolic Steroids and Sarm’s. *Sports Med Open*.

[41] Daher J., El Khoury D., Dwyer J. J. M. (2021). Education Interventions to Improve Knowledge, Beliefs, Intentions and Practices With Respect to Dietary Supplements and Doping Substances: A Narrative Review. *Nutrients*.

[42] Sharif S., Fergus S., Guirguis A., Smeeton N., Schifano F. (2024). Exploring the Understanding, Source of Availability and Level of Access of Cognitive Enhancers Among University Students in the United Arab Emirates: A Qualitative Study. *Human Psychopharmacology: Clinical and Experimental*.

[43] Wang Y., McKee M., Torbica A., Stuckler D. (2019). Systematic Literature Review on the Spread of Health-Related Misinformation on Social Media. *Social Science & Medicine*.

[44] Patel V., Aggarwal K., Dhawan A. (2024). Protein Supplementation: The Double-Edged Sword. *Proceedings (Baylor University. Medical Center)*.

[45] Rodriguez N. R., DiMarco N. M., Langley S. (2009). Position of the American Dietetic Association, Dietitians of Canada, and the American College of Sports Medicine: Nutrition and Athletic Performance. *Journal of the American Dietetic Association*.

[46] Kato H., Suzuki K., Bannai M., Moore D. R. (2016). Protein Requirements Are Elevated in Endurance Athletes After Exercise as Determined by the Indicator Amino Acid Oxidation Method. *PLoS One*.

[47] Samal J. R. K., Samal I. R. (2018). Protein Supplements: Pros and Cons. *Journal of Dietary Supplements*.

