# Supplementary material for: Assessment of the University Students' Knowledge, Attitudes, and Use of Protein Supplements: A Cross-Sectional Study From the United Arab Emirates
Source: J Nutr Metab. 2025 Nov 21;2025:5582105. doi: 10.1155/jnme/5582105 (PMC12662688; doi:10.1155/jnme/5582105)
Supplement: Supporting Information — Additional supporting information can be found online in the Supporting Information section. [file 5582105.f1.pdf]

Assessment of the University Students' Knowledge, Attitudes, and Use of Protein Supplements. A Cross-Sectional Study from the United Arab Emirates.

Do you agree to participate in this study and to use the information for scientific research purposes?

Yes

No

What is your gender?

Male

Female

In which age group do you belong?

18-25years

26-30 years

Above 30+

Do you go to sports clubs/gym regularly?

Yes

No

How many times do you visit the gym per week?

I don't go to the gym.

1-2 per week

3-5 per week

More than 5

Do you calculate your daily need for protein?

Yes

No

Do you take any protein supplements?

Yes

No

If you consume protein supplement, what kind of protein supplement do you take?

Powder

Protein Bars

Other Snacks:

(Brownies, Pancakes, cookies, chips)

If you consume protein supplement, when do you take protein supplements?

Before workout

Early morning

Right after workout

Other time

If you are using powdered protein, how many scoops (grams) do you consume daily?

½ - 1 scoop (12-24g)

1 scoop (24g)

1-2 scoops (24-48g)

More than 2 scoops

For what reason do you take protein supplements?

Building a muscular body

Gaining power and strength

Competitive advantage.

Improve body shape.

Others

Are there risks associated with taking protein supplements?

NO risk

Yes, but I don't know the risk

Kidney damage

Dehydration

Gout (Joint inflammation)

others

Are there benefits associated with taking protein supplements

No

I don't know.

Yes, but I don't know the exact benefits.

Building muscular body

Gaining power and strength

Improves performance.

Others

Who encouraged you to take protein supplements?

Friends/Relatives

Coach

Health Care Provider

Social media/Internet

Others

No one

Have you experienced any of the following symptoms while using protein supplements?

Diarrhoea

Indigestion

Nausea

Losing appetite

Constipation

Rashes (inflammation of the skin)

Itching

Stress

Insomnia

Menopause

Delayed menstruation

Headache

Vomiting

No symptoms

Others

Give us your opinion according to your knowledge:

Protein supplements are better than protein rich food to build muscles.

I agree

I strongly agree

Neutral

I disagree

I strongly disagree

Gym attendees should take protein supplements.

I agree

I strongly agree

Neutral

I disagree

I strongly disagree

Protein supplements are a good source of energy during exercise.

I agree

I strongly agree

Neutral

I disagree

I strongly disagree

To gain more muscles I need to take protein supplements.

I agree

I strongly agree

Neutral

I disagree

I strongly disagree

Taking Protein supplements minimize the accumulation of unwanted body fat

I agree

I strongly agree

Neutral

I disagree

I strongly disagree

Most people my age fulfil their protein needs.

I agree

I strongly agree

Neutral

I disagree

I strongly disagree

Eating large amounts of protein will negatively affect my health.

I agree

I strongly agree

Neutral

I disagree

I strongly disagree

Protein supplements are essential for building muscles through weight- lifting

I agree

I strongly agree

Neutral

I disagree

I strongly disagree
